# Cellular dynamics shape recombination frequency in coronaviruses

**DOI:** 10.1371/journal.ppat.1012596

**Published:** 2024-09-27

**Authors:** Cassandra M. Bonavita, Heather L. Wells, Simon J. Anthony

**Affiliations:** Department of Pathology, Microbiology, and Immunology, University of California Davis School of Veterinary Medicine, Davis, California, United States of America; Fred Hutchinson Cancer Center, UNITED STATES OF AMERICA

## Abstract

Coronavirus genomes have evolutionary histories shaped extensively by recombination. Yet, how often recombination occurs at a cellular level, or the factors that regulate recombination rates, are poorly understood. Utilizing experimental co-infections with pairs of genetically distinct coronaviruses, we found that recombination is both frequent and rare during coinfection. Recombination occurred in every instance of co-infection yet resulted in relatively few recombinant RNAs. By integrating a discrete-time Susceptible-Infected-Removed (SIR) model, we found that rates of recombination are determined primarily by rates of cellular co-infection, rather than other possible barriers such as RNA compartmentalization. By staggering the order and timing of infection with each virus we also found that rates of co-infection are themselves heavily influenced by genetic and ecological mechanisms, including superinfection exclusion and the relative fitness of competing viruses. Our study highlights recombination as a potent yet regulated force: frequent enough to ensure a steady influx of genetic variation but also infrequent enough to maintain genomic integrity. As recombination is thought to be an important driver of host-switching and disease emergence, our study provides new insights into the factors that regulate coronavirus recombination and evolution more broadly.

## Author summary

Recombination is a key driver of coronavirus evolution and disease emergence. However, our understanding of this mechanism or the factors that regulate it is extremely limited. Despite advances in sequencing technology, experimental studies on the frequency and drivers of recombination have been sparse. In fact, this issue hasn’t been closely studied in over 30 years. Our study takes a novel look at the factors that influence the rate of coronavirus recombination. By integrating experimental data with mathematical models, we show that the timing and order of viral infections significantly impact recombination rates. These findings shed light on the ecological and cellular dynamics that govern recombination, offering new insights into how coronaviruses evolve and adapt. By understanding the conditions that drive recombination, we can better predict the emergence of new viral strains, which is critical for forecasting spillover events and mitigating future outbreaks. Our study underscores the importance of recombination as a key process in viral evolution and highlights the need for continued research to fully understand its role in disease emergence.

## Introduction

Recombination is the process by which genetic material is rearranged to create new combinations of genes. For coronaviruses, it is both an integral part of the viral life cycle and a fundamental driver of genetic variation [1–4]. Recombination can occur in two ways: *intra*-molecular recombination, which involves genetic rearrangements within a single RNA molecule, and *inter*-molecular recombination, which involves the exchange of genetic material between separate RNA molecules, often leading to the formation of recombinant genomes.

Intra-molecular recombination occurs as a normal process during coronavirus transcription, where the viral polymerase dissociates from an RNA template and reattaches at a different position on the same molecule. This phenomenon takes place during negative-strand synthesis, particularly at transcription regulatory sequences (TRSs), where the viral polymerase disassociates and then reassociates upstream with the 5’ leader TRS in the untranslated region (UTR) [1,5]. A TRS precedes nearly every gene in the coronavirus genome, resulting in the production of a nested set of sub-genomic RNA (sgRNA) that regulate protein translation. Occasionally, this recombination process occurs at sites other than TRSs, leading to the formation of RNA molecules termed ‘defective RNA’ (dRNA), ‘non-canonical subgenomic RNA’ (nc-sgRNA), or sometimes ‘defective viral genomes’ (DVGs) [6–9]. These molecules exhibit significant structural variation in their sequence and typically lack the ability to generate functional virions, although they have been implicated in pathogenesis [10–12].

During inter-molecular recombination, the polymerase reassociates with a different RNA template. If the polymerase reassociates at the same genomic position on the second template, the recombination is referred to as ‘homologous’. Conversely, if the polymerase reassociates at a different position, the recombination is termed ‘non-homologous’. In singly infected cells, inter-molecular recombination may serve critical evolutionary roles by combining potentially advantageous mutations from multiple individual RNAs into a single template or by eliminating deleterious mutations [13]. The specific mechanism underlying inter-molecular recombination is not known; however, the leading hypothesis is that of ‘copy-choice’ recombination, where template switching occurs when the polymerase encounters a region of complementary sequence or secondary structure between two RNA molecules [3,13–15].

Occasionally, two different coronaviruses may infect the same cell, providing opportunity for *inter-typic* recombination to occur between genetically distinct viruses. Recombination between diverse genomes can promote phenotype shuffling and allow coronaviruses to rapidly explore new evolutionary space, potentially producing viruses with new combinations of traits that can affect transmissibility, pathogenicity, tropism, or antigenic escape [16,17]. While most instances of genetic shuffling likely yield combinations of traits that are deleterious, the numerous examples of recombinant viruses found in nature suggest that recombination does confer fitness advantages with some regularity. Whether the actual mechanism of recombination was evolutionarily selected as a means to rapidly generate new genetic diversity (e.g., as a method of ‘sexual selection’) or whether it arose as a byproduct of a lifecycle that requires the generation of subgenomic RNA templates for the regulation of protein translation is unknown [2]. However, inter-typic recombination provides an opportunity for coronaviruses to explore extensive evolutionary changes in short periods of time, and its ubiquity in nature suggests that it is often successful in this regard.

Almost all known human coronaviruses have evidence of recombination in their genomes. Both SARS-CoV-1 and MERS-CoV are recombinant viruses [18–21]. Recombination between variants of SARS-CoV-2 has also occurred over the course of the COVID-19 pandemic [22,23], and all of the coronaviruses that cause seasonal common colds in humans have evidence of recombination in their genetic history (HCoV-229E [24], HCoV-NL63 [24,25], HCoV-HKU1 [26], and HCoV-OC43 [27]). Examples of recombination are equally common among coronaviruses known to infect domestic animals, such as swine enteric coronavirus in pigs [28] and infectious bronchitis virus in poultry [29,30]. Multiple strains of canine coronavirus (CaCoV) and feline enteric coronavirus (FeCoV) have recombined with other existing CaCoV and FeCoV strains [31,32] and even with transmissible gastroenteritis virus (TGEV), a virus that infects pigs [33]. Recently, a new recombinant FeCoV emerged in Cyprus and has caused devastating impacts on local cat populations [34], demonstrating that recombinant viruses can have vastly different pathogenicity and virulence compared to their parents and pose severe risks to both human and animal health.

Although recombination is a frequent occurrence during normal coronavirus replication and is common between different coronaviruses in nature, it is unclear with what frequency two genetically distinct coronaviruses will recombine, if given the opportunity. Similarly, the genetic or ecological factors that regulate the rate of recombination are not well understood. Estimates of the frequency of recombination could be made by examining the prevalence of recombinant viruses in nature, but this rate would be a vast underestimate due to our inability to observe recombinants that were lost to drift or selection or were simply not sampled [35]. Ultimately, the true rate of recombination can only be ascertained through experimental studies [35]. Of the few such studies that exist, the majority were conducted several decades ago–long before current high-throughput sequencing technologies were developed. These early studies were also limited by using very closely related strains of the same virus, either avian infectious bronchitis (IBV) virus or murine hepatitis virus (MHV) [36–41], and did not examine recombination between genetically distinct viruses. A more recent experimental study by Gribble et al. [6] investigated patterns of *intra*-typic recombination for singly infected cultures of SARS-CoV-2, MERS-CoV, and MHV, but to our knowledge no modern studies have examined *inter*-typic recombination between two different parental coronaviruses.

Here, to examine the frequency of recombination between genetically distinct coronaviruses, we performed a series of co-infection experiments between CaCoV and FeCoV. These viruses were chosen because they are known to have recombined multiple times in nature [31,32,34], they are easily culturable in the same cell type, and they are BSL-2 level pathogens. As CaCoV and FeCoV are alphacoronaviruses, we also repeated the experiments with two betacoronaviruses, MHV and rat coronavirus (RCoV). We found that recombination occurs readily in vitro, with evidence of recombinant RNAs in every experiment performed. However, we also found that the number of recombinant RNAs produced is low. By integrating a cellular-level Susceptible-Infectious-Removed (SIR) model, we found that co-infection is a primary determinant of the rates of recombination. Further, by staggering the order and timing of infection with each virus, we found that the rates of co-infection and recombination are influenced by mechanisms of superinfection exclusion and the relative fitness of competing viruses. Our study makes long overdue and important advancements in our understanding of coronavirus recombination by 1), examining rates of recombination between genetically distinct viruses, 2) leveraging advancements in sequencing technology that allow the measurement of recombination at the level of individual RNA molecules, and 3), by integrating experimental and mathematical models to elucidate the factors regulating this critical component of coronavirus evolution.

## Results

### Recombination occurs consistently across co-infection experiments

Canine A72 cells were co-infected with CaCoV (strain 1–71 [42]) and FeCoV (strain WSU 79–1683 [43]) at a total MOI of 1 (0.5 of each virus). At 48 hours post-infection (hpi), cultures were chemically inactivated. Total RNA was then extracted, with polyA-tail selection, and sequenced using the Oxford Nanopore Direct RNA kit (see *Methods*). This experiment was repeated 9 times. On average across these experiments, 18% of all sequenced reads were viral, 42% were host, and the remainder were either yeast enolase 2, which serves as an internal positive control in the Direct RNA sequencing kit, or unclassified. Reads were screened with CZ ID [44] (www.czid.org) as a general screen for contamination, but no evidence of contamination was found. Both CaCoV and FeCoV genomes were sequenced with >99.9% coverage in all experiments, with an average depth of 1,311X and 1,241X, respectively (S1A Fig in S1 File). As expected, a large sequencing bias towards the 3’ end of the genome was observed, which is due to both the nested nature of sgRNAs as well as the 3’ bias of polyA tail RNA selection. The average viral read length was 1,875bp, with many full-length genomic RNA (gRNA) molecules detected that approached 30kb in length (S1B Fig in S1 File).

Each viral read exceeding a minimum size of 1,000 bp was classified using a custom likelihood-based sorting algorithm we called *NanoSort*. Validation of *NanoSort* was performed using simulated recombinant reads and resulted in a sensitivity of 98.6% for recombination detection (S2 Fig in S1 File). A minimum read size of 1,000 bp was imposed as *NanoSort* requires a minimum of 500 bp from each parental sequence to call a read recombinant, making the minimum detectable size of recombinant RNA 1,000 bp (see *Methods*). Reads exceeding this threshold were classified as sgRNA, structural variant RNA (e.g., dRNA, nc-sgRNA, or DVGs), recombinant RNA, or none of the above. We note that while sgRNA and structural variant RNA are presumed to be generated via a mechanism of non-homologous recombination, for simplicity here ‘recombinant’ refers specifically to molecules that have sequence origins from both parental viruses only.

**Fig 1:**
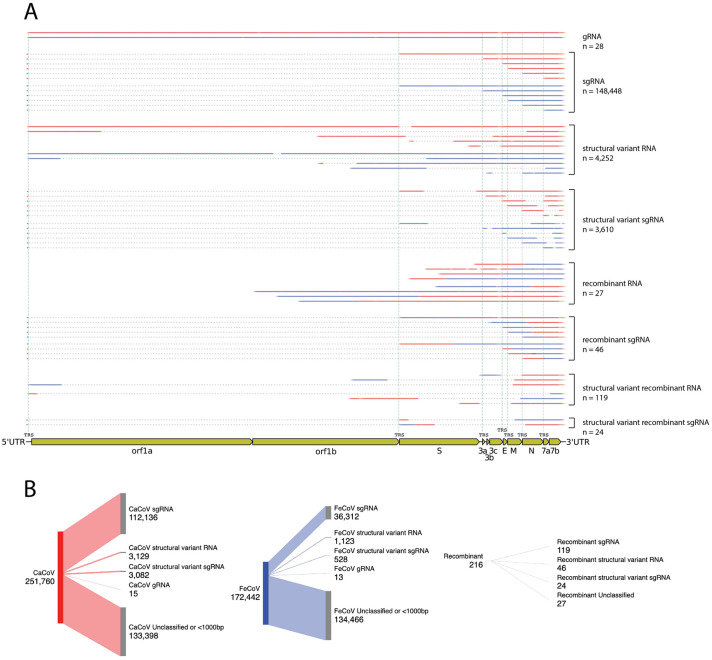
Recombination occurs and is detectable alongside sgRNA and structural variant RNA in co-infection experiments. A) Examples of RNA molecules detected during a single co-infection experiment between CaCoV strain 1–71 and FeCoV strain WSU-79-1683 are shown. The experiment was performed with a starting MOI of 1 (0.5 per virus) and extracted and sequenced 48 hours post-infection. Each type of RNA molecule is aligned to the CaCoV genome (gene boundaries annotated in yellow, below) and colored according to the parental origin of the nucleotide sequence (red: CaCoV, blue: FeCoV). Dotted lines indicate deleted sequence. Vertical green dashed lines indicate positions of genomic transcription regulatory sequences (TRSs). B) Sankey diagrams of RNA molecule counts for reads assigned to CaCoV, FeCoV, or as recombinant. Made at SankeyMATIC.com. Categories of RNA molecules are abbreviated in both panels as: gRNA: genomic RNA, sgRNA: subgenomic RNA.

The vast majority of the reads in each experiment were non-recombinant and derived from a single parental virus (>99% of viral reads). For each parent, all RNAs expected to be produced during natural infection were identified, including full-length gRNA, all canonical sgRNAs, and numerous structural variants (Fig 1). Reads were often classified into more than one category, with many examples identified of sgRNAs that had downstream structural variants within them or also showed evidence of recombination between parents. Structural variant reads where one side of the junction came from one parent and the other side of the junction came from the other parent were also identified. In a few cases, reads were classified into all three categories.

**Fig 2:**
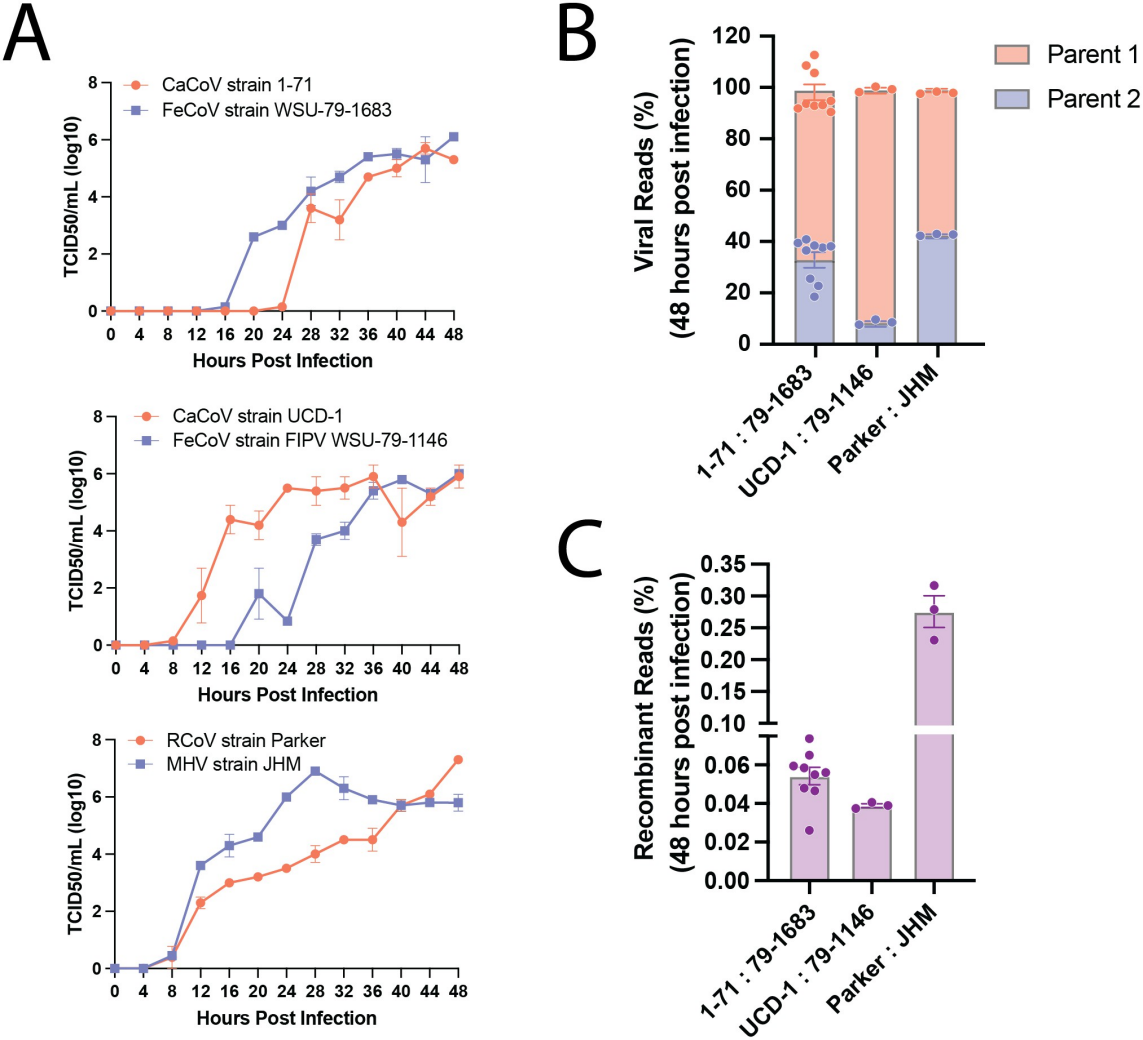
Different pairs of coronaviruses exhibit different replication dynamics, proportions of parental reads, and rates of recombination. (A) Viral replication kinetics measured as TCID_50_ were evaluated in duplicate every 4 hours for 48 hours. All infections were conducted individually at an MOI of 0.1. CaCoV and FeCoV infections were conducted in A72 cells and RCoV and MHV infections were conducted in L2P41.a cells. (B) Percentages of total viral reads assigned to each parental virus detected for all three pairs of co-infected viruses. (C) Percentages of recombinant reads with origins from both parental viruses detected for all three pairs of co-infected viruses. For panels B and C, each experimental replicate is shown as a point. Bars represent the mean of all replicates and error bars represent the standard error of the mean for each group. All experiments in B and C were performed at a total MOI of 1 (0.5 per virus) and extracted after 48 hours.

We also measured the overall proportion of CaCoV and FeCoV reads in each experiment. The more evenly distributed the two viruses are in the infection, presumably the higher the opportunity for them to co-infect the same cell. The distribution of parental reads was slightly imbalanced, with 65% of viral reads coming from CaCoV and 35% coming from FeCoV (Fig 2B). This bias was not unexpected, as competitive fitness differences can emerge during co-infection scenarios. When CaCoV and FeCoV were cultured individually, they showed similar replication kinetics and end point titers, with the exception of an initial lag for CaCoV (Fig 2A). Despite this lag, CaCoV produced more viral RNAs than FeCoV, revealing a competitive advantage during co-infection of canine A72 cells.

Despite the slight bias in favor of CaCoV, many recombinant RNAs between the two parental viruses were identified (Fig 1). Interestingly, recombinant RNAs were detected across all 9 replicates. To quantify the rate of recombination in each experiment, we opted for a simple measure of the number of recombinant RNA molecules produced relative to the total number of viral RNA molecules that exceeded 1,000 bp. While evidence of recombination was observed consistently in every experiment, on average the per-molecule recombination rate was only 0.05% of all viral reads observed (Fig 2C). We also calculated the per-nucleotide recombination rate as the number of recombination events observed divided by the total number of nucleotides sequenced from reads greater than 1,000 bp, which on average was 2.46x10^-7^ recombination events per nucleotide.

As a confirmation of the authenticity of detected recombinants, we ran a series of negative control experiments. First, we conducted mock or singly infected CaCoV or FeCoV experiments at an MOI of 2 for 48 hours. As expected, there were no viral reads observed in the mock infected cells. In the single infections, >99% of viral reads correctly mapped to the input virus, with only an extremely marginal percentage (0.0004% and 0.007% respectively) aligning to the incorrect virus (representing the slight error involved in using read mapping to classify whole reads). Notably, no recombinant reads were observed in either infection. Next, to determine whether artificial chimeric reads would be generated during library preparation or sequencing, CaCoV and FeCoV were infected singly at a total MOI of 2 for 48 hours and then combined at three different steps of the library preparation and sequencing process: RNA extraction, polyA selection, and library preparation. This design emulated co-infection experiments without allowing CaCoV and FeCoV to co-replicate, which should result in no observed recombination. We observed 0 recombinant reads in the control combined after extraction, 9 (0.0067%) recombinant reads in the control combined after polyA selection, and 10 (0.0023%) recombinant reads in the control combined following library preparation. While this does indicate that some false positive recombinants were identified in our mixed controls, the rate was 10-fold lower than what was observed for experimental co-infections and does not significantly impact our findings. Notably, the recombinants observed in these two experiments were also highly redundant, most likely arising from misidentification of non-recombinant reads as false positive recombinant reads by *NanoSort* and not chimeric molecules.

### Different pairs of coronaviruses exhibit different rates of recombination

To evaluate whether our results were strain-specific, we additionally tested a second pair of CaCoV/FeCoV strains, CaCoV UCD-1 and FeCoV (FIPV) WSU-79-1146. CaCoV UCD-1 is a serotype 2B virus, while CaCoV 1–71 is serotype 2A. Both FeCoV strains are serotype 2A, but FeCoV WSU-79-1146 is a strain of feline infectious peritonitis virus (FIPV), which has altered pathogenicity despite its sequence similarity (S3 Fig in S1 File). This time the experiment was repeated 3 times and recombination was similarly detected in all 3 replicates. Again, the rate of recombination was low, at 0.04% of viral reads (Fig 2C). For this pair, the bias towards CaCoV was even more pronounced, with >90% of the viral reads classified as CaCoV (Fig 2B). Finally, we extended these experiments to a pair of betacoronaviruses to evaluate whether the trend would be maintained in a different genus. We selected MHV (strain JHM) and RCoV (strain Parker) because they both replicate similarly in L2P41.a cells (Fig 2A). Again, 3 replicates were performed, and again recombination was observed in every experiment. The average rate of recombination for this pair was higher at 0.28%, and there was less bias in the proportions of reads classified to each parent.

Together, these results demonstrate that recombination occurs frequently, in the sense that it was detected in every experiment, but also infrequently in the sense that the proportion of recombinant RNAs produced is low. In general, the more even the distribution of parental virus RNA, the higher the observed recombination rate. MHV-JHM and RCoV-Parker had the most even distribution of parental virus RNA after 48 hours (~43% and 57%, respectively) and had the highest average rate of recombination (0.28%). Conversely, the least comparable pair (CaCoV UCD-1 and FeCoV WSU-79-1146, ~91% and 9% respectively) also had the lowest recombination rate (0.039%). When one virus is in excess, there can be fewer cells that are co-infected with both viruses at the same time, and thus less opportunities for recombination. This led to the conclusion that differences in our observations across virus pairs may be driven by different rates of cellular co-infection for each virus pair.

### Recombination rate is variable depending on experimental conditions

If indeed recombination was being driven by rates of co-infection, we expected that varying experimental conditions that would change the rates of co-infection would also change the rates of recombination. By infecting at higher MOI, we suspected that a higher proportion of cells would be co-infected at onset and result in higher rates of recombination. We also suspected that longer co-replication times would allow more time for co-infection to occur and for recombinants to accumulate. Using CaCoV strain 1–71 and FeCoV strain WSU-79-1683, we next performed a series of the same co-infection experiments with different starting total MOIs (0.1, 1, 2, 5, and 10) and different co-replication times (harvesting at 12, 24, and 48 hpi). For each combination of MOI and co-replication time, we performed 3 replicates, resulting in a total of 45 experiments.

**Fig 3:**
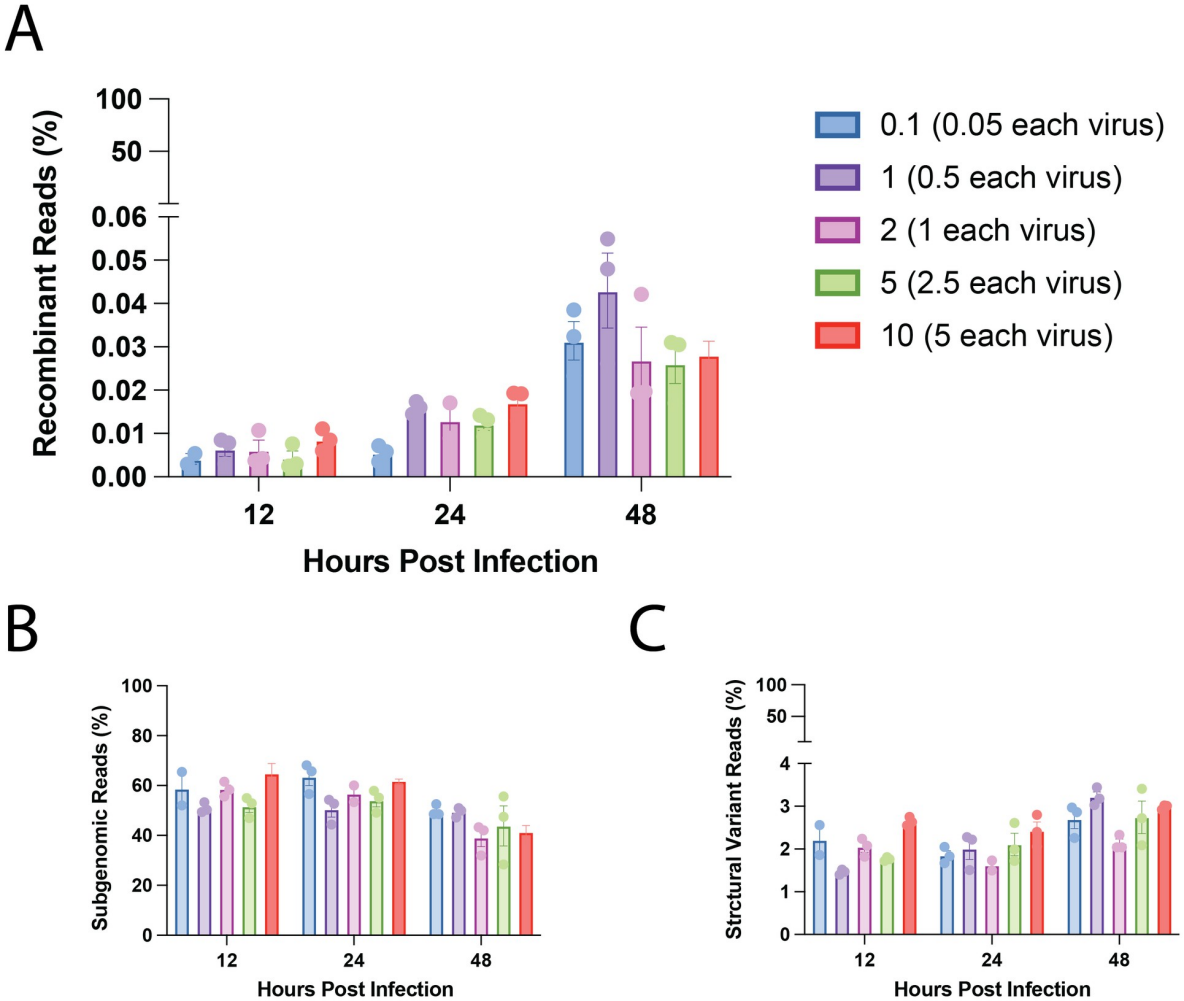
Recombination rate is variable depending on experimental conditions. (A) Percentages of recombinant reads, (B) percentages of subgenomic reads, and (C) percentages of structural variant reads observed across all experimental conditions for co-infections with CaCoV strain 1–71 and FeCoV strain WSU-79-1683. For all panels, colors correspond to starting MOI (upper right) and extraction times are grouped on the x-axis. Each experimental replicate is shown as a point. Bars represent the mean of all replicates and error bars represent the standard error of the mean for each group. All experiments were performed in triplicate and extracted at either 12-, 24-, or 48-hours post-infection with total starting MOIs of 0.1, 1, 2, 5, or 10. Only the first three replicates for MOI of 1 at 48 hpi are shown.

In agreement with our initial co-infection experiments, we observed recombinant reads in each of the 45 experiments. However, the rates of recombination varied considerably across the different experimental conditions (Fig 3A). In general, longer co-replication times resulted in higher rates of recombination as expected, but increasing MOI did not necessarily increase rates of recombination for the same co-replication time. Instead, rates of recombination seemed to follow a non-linear shape with MOI at each time point, increasing from MOI of 0.1 to MOI of 1 before decreasing at MOI of 2 and 5, then increasing again at MOI of 10. Conversely, rates of non-recombinant sgRNA production appeared much more consistent across all experimental conditions (Fig 3B), with rates around 45–65%, which is consistent with rates of sgRNAs reported for other coronaviruses [45]. Rates of structural variant production averaged around 1.5–3% and increased only slightly with increasing co-replication time (Fig 3C), which is also consistent with trends reported for other coronaviruses [46]. Results of read classifications for all sequencing runs reported in this study are tabulated in S2 File.

Interestingly, we also found that the diversity of the recombinant reads observed in each experiment decreased over time. That is, at earlier time points, the majority (if not all) observed recombinant reads were unique, while at later timepoints, the same recombinant was occasionally observed more than once. We used Shannon’s diversity index to calculate the diversity of recombinant reads for each experiment and standardized this measure relative to the maximum value obtained when all observed recombinants were unique. Thus, when all observed recombinants in an experiment were unique, this value was 1, and when some recombinants in the experiment were observed more than once, this value decreased from one (indicating decreased diversity). We found that the relative diversity index decreased as the length of the experiments increased, but we did not observe any relationship with starting MOI (S4 Fig in S1 File). The relative diversity index for each experiment is reported in S2 File.

**Fig 4:**
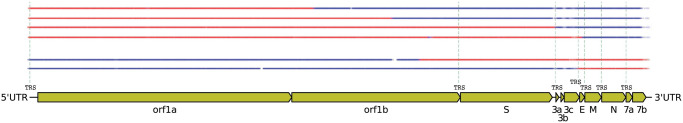
Genome-length recombinant RNA observed across all experimental replicates. Six instances of genome-length recombinant reads were observed across all CaCoV 1–71 and FeCoV WSU-79-1683 experiments and replicates. As in Fig 1, each type of RNA molecule is aligned to the CaCoV genome (gene boundaries annotated in yellow, below) and colored according to the parental origin of the nucleotide sequence (red: CaCoV, blue: FeCoV). Dotted lines indicate deleted sequence. Vertical green dashed lines indicate positions of genomic transcription regulatory sequences (TRSs).

Across all co-infection experiments performed with CaCoV 1–71 and FeCoV WSU-79-1683 (including the 45 experiments shown here as well as the 9 at MOI of 1 and 48 hpi shown in Fig 2), it is also notable that we observed several instances of recombinant reads that were full-length (Fig 4). Additionally, each full-length recombinant RNA observed was unique, and the same molecule was never observed twice. It is not known whether these full-length recombinant RNAs could serve as functional gRNA and generate infectious recombinant virions with continued propagation, as it is unclear whether any complex genomic regulatory regions or epistatic interactions were disrupted. However, the observation of intact, genome-length recombinant RNA is a promising finding that the production of infectious recombinant virions may be occurring in these experiments.

### Predicted dynamics of cellular co-infection are reflected in observed recombination rates

**Fig 5:**
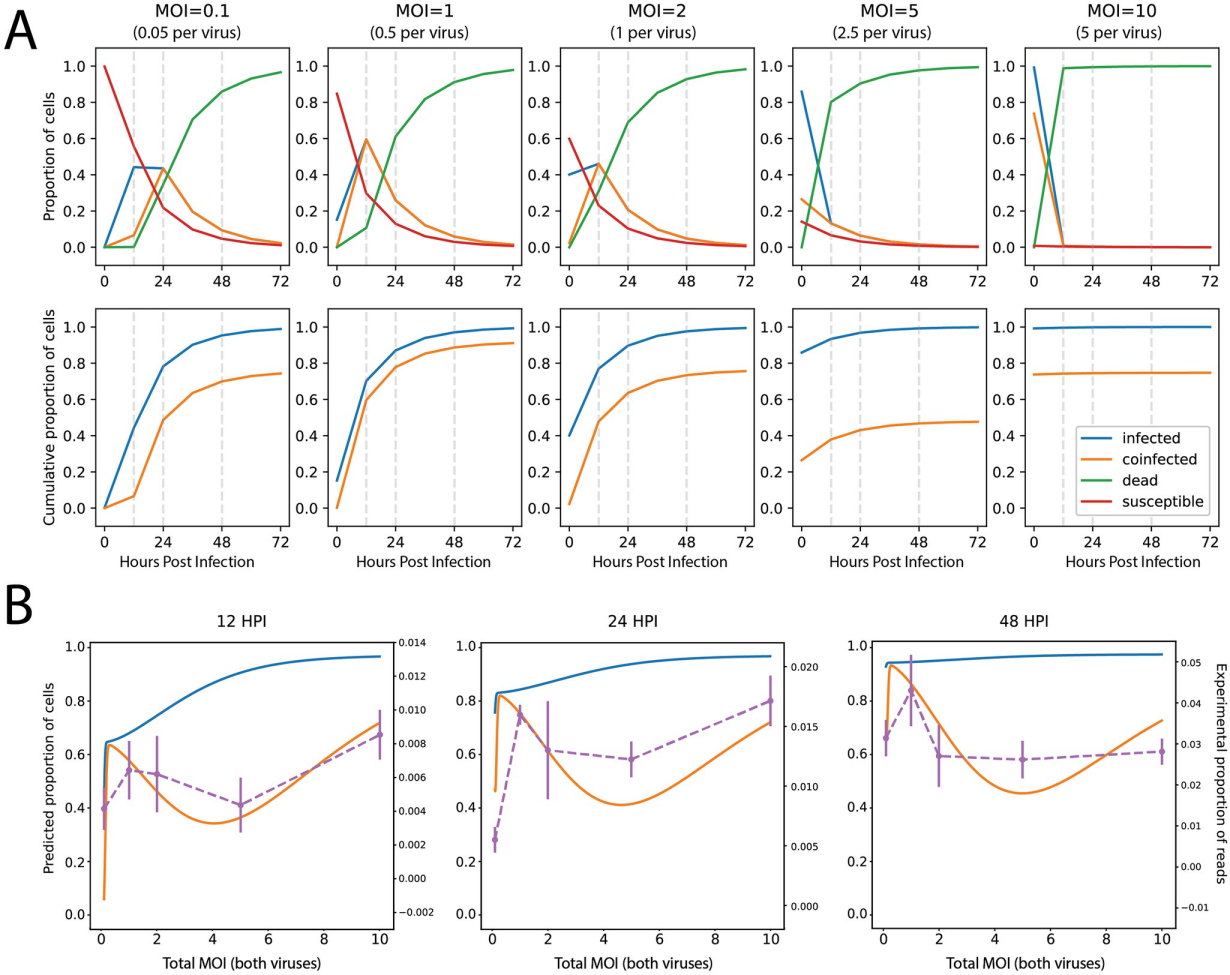
Predicted dynamics of cellular co-infection across time at different starting MOI are reflected in observed recombination rates. (A) Above: proportions of cells that are susceptible (red), singly infected (blue), co-infected (orange), or dead (green) predicted at 12-hour time intervals post-infection by a discrete-time SIR model. Below: cumulative proportions of cells either infected (blue) or co-infected (orange) predicted at 12-hour intervals over the course of the infection. Each column represents predictions made at different starting MOI. Vertical dashed lines are plotted at 12-hour intervals. (B) Predicted cumulative proportions of cells either infected (blue) or co-infected (orange) predicted as a function of MOI. Each panel represents predictions made at different time points post-infection.

We suspected that the non-linear trend in recombination rates with increasing MOI could be explained by infection dynamics that affect co-infection rates across time. In order to test this hypothesis, we developed a model that could be used to estimate the proportion of cells expected to be co-infected across these different conditions to determine if this could explain the observed recombination rates. We chose to model cellular-level infection dynamics using a discrete-time Susceptible-Infectious-Removed (SIR) model (Fig 5A), from which we could examine patterns of co-infection across different values of both co-replication time and initial MOI. As expected, we observed that a higher proportion of cells are predicted to be co-infected at onset at high starting MOI compared to low MOI and that with increasing co-replication time, the cumulative proportion of co-infected cells increases under all conditions. However, infected cells die after a period of time, resulting in fewer remaining susceptible cells to be infected during subsequent replication cycles. For medium to high starting MOI, such as 2, 5 or 10, the majority of the cells are infected at onset and die early in the experiment. At MOI of 2 or 5, the majority of cells are *infected* at onset but not necessarily *co-infected*, resulting in the majority of cells dying before having the opportunity to become co-infected and with few susceptible cells remaining to sustain this dynamic. Only at an MOI of 10 are the vast majority of cells co-infected at onset, but they die early in the experiment and the infection is not sustained over time. Conversely, for lower starting MOI such as 0.1 or 1, the proportion of cells predicted to be co-infected at onset is low, but additional co-infected cells accumulate in subsequent replication cycles, eventually outpacing the proportion co-infected at onset for higher starting MOI.

Using predictions from our SIR model, we plotted the expected cumulative proportion of co-infected cells at 12, 24, and 48 hpi as a function of input MOI and found that the dynamics of co-infection across different MOIs was highly non-linear (Fig 5B). We then overlaid our observed rates of recombination with the SIR model predictions and found that the trends were highly consistent: the rates of co-infection predicted by the SIR model across varying input MOIs and co-replication times followed the same trend as the recombination rates observed in our experimental data, confirming our suspicion that rates of recombination are driven by rates of cellular co-infection.

### Order and timing of virus infection also alter cellular co-infection and recombination rates

**Fig 6:**
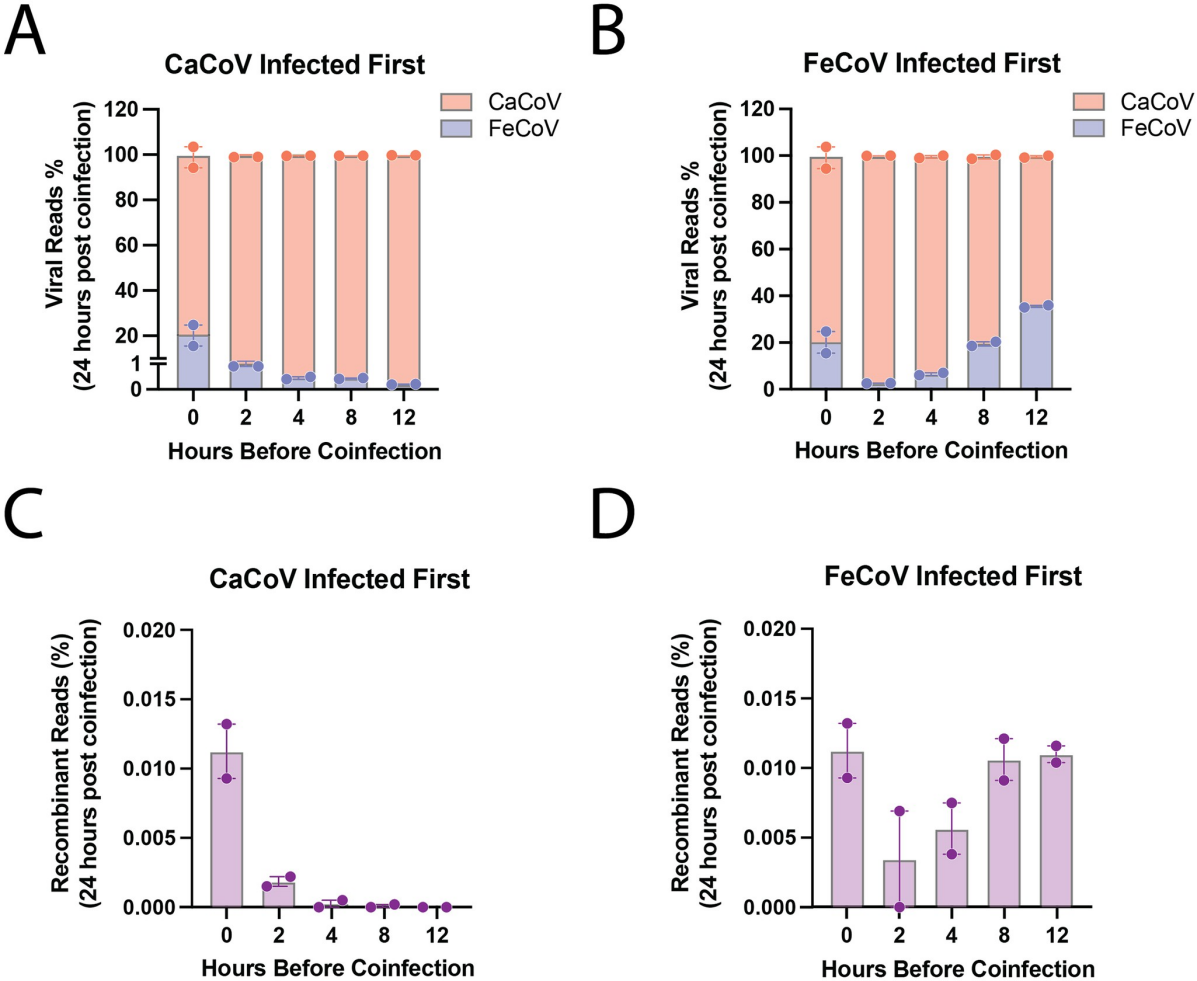
Order and timing of virus infection also alter cellular co-infection and recombination rates. (A) and (B): Percentages of total viral reads assigned to CaCoV strain 1–71 and FeCoV strain WSU-79-1683 24 hours after co-infection where either CaCoV (A) or FeCoV (B) were infected first and allowed to establish for 2, 4, 8, or 12 hours before co-infection with the second virus. (C) and (D): Percentages of recombinant reads detected in the same staggered experiments as panels (A) and (B). Non-staggered data is included from the co-infections at total MOI of 5 from previous experiments as a baseline.

We similarly suspected that rates of cellular co-infection would be impacted by the order and timing of infection and would further modulate rates of recombination. We therefore repeated our experiments by staggering co-infection times by 0 (control), 2, 4, 8, or 12 hours to simulate delayed arrival of the second virus. We performed these experiments using CaCoV strain 1–71 and FeCoV strain WSU-79-1683. Two replicates were performed for each time point, for both sequences of infection (i.e., CaCoV first, then FeCoV first), totaling 16 distinct experiments. An MOI of 5 (2.5 per virus) was used to ensure the majority cells were infected with the first virus and that the second virus would be forced to infect a previously infected cell (i.e., superinfection). The distribution of viral reads between CaCoV and FeCoV and the rate of recombination were quantified for each scenario (Fig 6). Our data revealed a strong dependence of both the viral distribution and recombination rates on the timing and sequence of infection. Notably, when CaCoV was the initiating virus, FeCoV struggled to establish a significant superinfection if introduced just 2 hours later. As the delay increased, the presence of FeCoV diminished further. In contrast, when FeCoV was the initial virus, CaCoV dominated the viral reads, even at the shortest delay, contrary to expectations. This dominance persisted, although the proportion of FeCoV reads incrementally increased with longer delays between infections. Interestingly, we noted that the rates of recombination were closely aligned with the shifting proportions of each virus: recombination rates dropped when the initial infecting virus dominated, but as the balance shifted with increased staggering times, so too did the recombination rates. These experiments additionally support that rates of recombination are driven by changes in rates of cellular co-infection. Moreover, they demonstrate that rates of co-infection and recombination are modified by ecological (e.g., the order and timing of virus infections) and cellular (e.g., superinfection exclusion) mechanisms.

## Discussion

Extensive genetic evidence shows that recombination is common in coronaviruses [21,47–51], including in CaCoV [33,52,53] and FeCoV [31,32,34]. However, the true rate of recombination—defined here as the frequency at which RNA molecules from two parental viruses recombine—has remained elusive due to a lack of experimental studies. Using pairs of different strains of CaCoV and FeCoV (Genus *Alphacoronavirus*), as well as MHV and RCoV (Genus *Betacoronavirus*), we discovered that in general, the rate of RNA recombination for these viruses is low. The average per molecule recombination rate (calculated as the number of recombinant molecules divided by the number of viral molecules) varied depending on the experimental conditions and viruses used. The majority of our experiments were performed with CaCoV 1–71 and FeCoV WSU-79-1683, and we observed an average per molecule recombination rate of 0.025% per experiment, with a minimum of 0.0025% and a maximum of 0.074%. As our rates are calculated across the length of the experiment, they are not directly comparable to rates of recombination *per replication cycle*. Experiments performed at 24 and 48 hours likely encompass multiple rounds of replication. The experiments performed at 12 hours are the closest estimate to a per replication cycle rate, which varied from 0.004% to 0.01% (Fig 5B). Comparisons across the three pairs of viruses tested (performed at MOI of 1 for 48 hours) show that CaCoV UCD-1 and FeCoV (FIPV) WSU-79-1146 had the lowest average rate of per molecule recombination (0.039%), followed by CaCoV 1–71 and FeCoV WSU-79-1683 (0.054%) and MHV/RCoV with the highest (0.28%) (Fig 2). In general, the per-nucleotide recombination rate (calculated as the number of recombination breakpoints observed divided by the total number of nucleotides sequenced) varied on the order of 10^−7^ to 10^−8^ events per nucleotide per experiment for CaCoV 1–71 and FeCoV WSU-79-1683. For comparison, the rate of mutation for coronaviruses is on the order of 10^−6^ mutations per nucleotide per cycle [54,55]. Rates for all experimental conditions and virus pairs are presented in S2 File.

There are several factors, however, that may make this rate an underestimate. First, given the fragility of RNA, it is possible that some of the molecules sequenced were fragmented. Given that RNA sequencing selects for molecules with a 3’ polyA tail, any recombination events on the 5’ end of a fragmented RNA would not be observed. Further, sequencing coverage is highly biased towards the 3’ end of the genome (S1 Fig in S1 File), producing an unavoidable observation bias against recombinants that may be occurring towards the 5’ end of the genome. Bias against the 5’ end of the genome would apply for both recombinant and non-recombinant RNA, however, and would not significantly affect the estimate of recombination unless the underlying rate of recombination varies between the 5’ and 3’ ends of the genome. Second, limitations of our detection method using *NanoSort* prevent the detection of recombinants in reads shorter than 1,000 base pairs or where the breakpoint is more than 500 base pairs from the end of the read. We have attempted to correct for this by limiting our denominator to only reads that exceed 1,000 base pairs, but we cannot account for the missed detection of breakpoints closer to the ends of reads or where a recombinant portion in the middle of a read is less than 500 base pairs. Third, the denominator of our rate may be artificially inflated by a high amount of parental RNA persisting from the original infection, even after 48 hours. This effect would be especially pronounced at high MOI. Counting only RNA molecules produced during the experiment and not persisting from onset would be a more accurate estimate of the rate of recombination per replication cycle instead of a rate observed across the length of the entire experiment.

We must also emphasize that the rate of recombination reported here is calculated using the number of recombinant *molecules* observed and does not represent the number of functional recombinant *viruses* that may have been generated during the experiments. There is limited evidence that any recombinant viruses were functionally replicating in our experiments, although the possibility that they were generated and remained at low frequency cannot be ruled out. We did observe six instances of genome-length recombinant RNA in our experiments, and presumably, an RNA molecule spanning the entire length of the genome could function as gRNA and be packaged into a budding virion. Given that we did not passage any co-infected material or perform plaque assays, it is unclear whether any recombinant RNA generated during our experiments could produce an independent and functional infectious virus. The observation of recombinant sgRNA in our experiments could also be indicative of replicating recombinant viruses but could also potentially be the result of *de novo* recombination during transcription of a non-recombinant parental virus. Given we did not observe any experiments where multiple nested sgRNAs contained the same recombination breakpoint, the latter case seems more likely.

In general, however, we did observe that the diversity of recombinant reads decreased the longer the experiment was allowed to continue (S4 Fig in S1 File), which could indicate that replication was indeed occurring on recombinant molecules. For shorter experiments (12 hour co-replication times), we found that almost every recombinant read observed was unique. When experiments were longer (24 to 48 hours co-replication time), we occasionally observed the same point of recombination in more than one read. It is unclear whether multiple recombination events at the same site occurred more than once independently, possibly indicating the presence of a recombination hotspot, or whether a single recombination event produced one recombinant RNA molecule that was subsequently replicated. Replication of a molecule does not necessarily mean a functional recombinant virus was replicating, however, as small, non-genome-length RNAs could also be serving as templates of replication. Thus, while the generation of functional recombinant viruses in our experiments is presumably possible, we did not observe strong evidence for or against its occurrence.

Despite the low overall rate of recombination, we did observe recombinant RNAs in every experiment we performed. This observation highlights a complex dynamic where recombination occurs consistently during co-infection but results in a relatively small proportion of recombinant RNAs (an even smaller proportion of which can generate functional recombinant viruses). This balance between frequent recombination events and a low yield of recombinant products aligns well with the theory that maintaining both stability and innovation is vital for viral survival [56–60]. Recombination can therefore be viewed as a conservative but opportunistic process—frequent enough to ensure a steady influx of genetic variation but infrequent enough to maintain genomic integrity. While excessive recombination would likely lead to deleterious genetic configurations (i.e., the error catastrophe), insufficient recombination would limit adaptability and the occasional breakthrough of novel strains with advantageous traits (i.e., the adaptation catastrophe) [58,61].

We note that the recombination rates observed here contrast with early work by Baric et al., who reported rates of up to 25% between temperature sensitive MHV mutants [62]. Differences in methodologies, viral systems, and the basis for how rates were calculated, complicates direct comparison with our results. However, most importantly our study focused on recombination between genetically distinct virus strains (i.e., CaCoV and FeCoV, or MHV and RCoV), while Baric et al. studied recombination within the same strain of MHV. Rates of recombination may be significantly modulated by the genetic relatedness of the two parental viruses involved. In support of this hypothesis, we found higher rates of recombination between strains that are more genetically similar (i.e., MHV and RCoV), though our results are currently confounded by the fact MHV and RCoV also showed a more even fitness during co-infection. Additional studies are therefore required to determine whether rates of recombination are influenced more by the genetic relatedness of the two parents or by the relative fitness of the co-infecting viruses.

The apparent paradox of recombination as a common outcome in nature and a rare event at the cellular level can, we suggest, be reconciled by the vast number of hosts that likely become infected by each virus over time. Both FeCoV and CaCoV are common infections of cats [63–66] and dogs [67,68], creating numerous opportunities for co-infection. Each recombination event harbors the potential for significant evolutionary leaps. Therefore, even if individual instances of co-infection yield few recombinants, the cumulative effect over numerous co-infections and the selective advantage of certain recombinants can nonetheless drive their prevalence in the population. Similar dynamics have been proposed to explain the emergence of reassortant influenza viruses [69]. Recombination, then, emerges as a potent yet regulated evolutionary process, where its rarity in individual co-infections preserves genomic integrity, while its collective impact over time enables the diversification of the viral genome and adaptation to changing environments. The numerous examples of CaCoV and FeCoV recombination in nature may well attest to the evolutionary efficacy of this balanced approach.

We found that rates of recombination are modified by ecological and cellular processes. Recombination events were observed across all unstaggered co-infection scenarios, and discrete-time SIR models and staggered co-infection experiments substantiated that these events are heavily contingent on co-infection rates. This implies that co-infection is a critical determinant of recombination and that other intracellular barriers, such as RNA compartmentalization (e.g., within different replication organelles) or genetic incompatibilities during template switching, do not substantially restrict recombination for the viruses evaluated here. Indeed, we found that if CaCoV and FeCoV co-exist within a single cell, recombination is a probable outcome. Critically, this implicates mechanisms that regulate co-infection rates as fundamental drivers of the evolutionary dynamics of these viruses.

One such mechanism is superinfection exclusion (SIE), which our findings suggest plays a significant role in modulating the likelihood of co-infection, and subsequently recombination. The many examples of recombination in nature support that superinfection must occur, especially given that most co-infections in nature are likely to be asynchronous. Direct evidence for superinfection has even been reported for variants of SARS-CoV-2 [23]. While superinfection itself is well-documented, the role of SIE, which prevents secondary infections from establishing within an already infected cell, has been less explored for coronaviruses. Indeed, while SIE has been demonstrated for many virus systems [70,71] and has been particularly well-studied in influenza virus [69,72], its role in coronaviruses dynamics and evolution is relatively under-studied.

Historical studies from the 1950s and 60s first hinted at interference mechanisms within coronaviruses, showing that infectious bronchitis virus (IBV; Genus *Gammacoronavirus*) could interfere with subsequent infection by Newcastle disease virus (NDV; Family *Paramyxoviridae*) [73–75]. However, these early studies did not explore SIE between coronaviruses, whether between identical viral particles of the same strain (e.g., SARS-CoV-2) or between genetically distinct lineages (e.g., CaCoV and FeCoV). More recently, direct evidence of SIE between isogenic strains of SARS-CoV-2 has emerged [76]. Sims provided compelling evidence for SIE by demonstrating that a short time-delay limits the ability of genetically identical viruses to superinfect the same cells, leading to spatially distinct viral populations [76]. Our study demonstrates that SIE also occurs between genetically distinct coronaviruses. Specifically, we found that when CaCoV is introduced to the cells first and given time to establish an infection before FeCoV is introduced, the ability of FeCoV to infect and establish itself is significantly reduced—a hallmark of SIE.

In the context of our study, where CaCoV often outcompetes FeCoV even when FeCoV infects first, we see that the dynamics of SIE are not only time-dependent but also tied to the inherent competitive and cooperative characteristics of the infecting viruses. Indeed, the interplay between these ecological and cellular mechanisms—specifically, the order of infection and the temporal window before superinfection—creates a complex regulatory system for recombination. When CaCoV infects first, its competitive dominance comes into play, inhibiting the secondary establishment of FeCoV and limiting co-infection and recombination rates compared to when the two viruses are infected at the same time. This is surprising given that FeCoV exhibited similar or slightly faster growth kinetics compared to CaCoV in A72 cells (Fig 2A); however, SIE may be an advantageous enough trait to overcome the limitations of slower growth rates [77]. In scenarios where FeCoV is infected first without CaCoV, FeCoV fails to establish a robust enough infection before CaCoV is introduced that is sufficient to produce co-infection and recombination rates compared to when the two viruses are infected at the same time. This suggests that the relationship between CaCoV and FeCoV may not be entirely antagonistic, as has been shown for other virus systems [78,79]. Only when CaCoV is introduced after 12 hours do we see that FeCoV has sufficient time to establish an infection, reducing the impact of SIE and consequently providing more opportunities for co-infection and recombination. These results are particularly significant given that, as mentioned above, in natural settings asynchronous infections are likely the norm and not the exception.

Our findings demonstrate that recombination is a potent yet regulated evolutionary force. The numerous examples of recombination reported in the literature, coupled with our observation that recombination occurs consistently during co-infection, supports that recombination occurs frequently. However, the relatively low number of recombinant RNAs produced and the mechanisms that limit opportunities for recombination to occur (e.g., superinfection exclusion) demonstrate that mechanisms also exist to regulate this process. We find that the combined effects of ecological factors, such as the order of infection, and cellular factors, such as superinfection exclusion, are likely major contributors to the evolutionary dynamics of coronaviruses. They determine not only the immediate fate of viral populations within a host but also the long-term evolutionary potential of these viruses as they spread and diversify across hosts and environments.

Our study also opens several lines of future inquiry. Foremost, this includes investigations into whether rates of recombination vary depending on the genetic relatedness of the parental viruses. Currently, the precise limitations of recombination between genetically distinct coronaviruses remain incompletely described. Conducting experimental studies involving co-infections across a spectrum of genetic similarity would provide valuable insights into this aspect. Additionally, while our observations indicate clear evidence of superinfection exclusion, the specific mechanism responsible for this phenomenon remains elusive. Exploring the mechanistic basis of superinfection exclusion would enhance our understanding of how ecological and cellular factors intersect to influence rates of recombination and, consequently, opportunities for virus evolution.

Finally, our recent delineation of the recombination pathway—a series of barriers preceding and succeeding the recombination event—paves the way for further investigations that could improve our ability to predict where and when new recombinants are likely to emerge [35]. These insights are fundamental for developing models that forecast the emergence of new coronaviruses in specific host species and geographic regions, guiding targeted surveillance for early detection of emerging recombinants at particular hotspots. While biosurveillance helps identify emerging recombinants, understanding the fundamental forces that drive their amplification and spread within these hotspots is also critical. One such mechanism to study is the fitness of recombinant viruses, which determines their ability to compete with parental strains and propagate through host populations. By studying the selective advantages or disadvantages conferred by recombination, we can better predict which recombinants are likely to propagate in mixed viral populations, overcoming competitive barriers with parental strains. This knowledge may allow us to anticipate the conditions under which new strains may emerge and dominate in both human and animal populations. Understanding this process is also critical for vaccine and therapeutic design, as recombination plays a key role in generating viral diversity and potentially producing variants that evade immunity. By studying how and when recombination occurs, we may be able to predict or even prevent the emergence of new strains that escape vaccines or develop drug resistance. For example, identifying conserved regions of the viral genome that are less likely to undergo recombination—or where recombination leads to non-viable variants—could guide the design of broad-spectrum vaccines targeting those stable regions, helping vaccines remain effective even as the virus evolves.

## Methods

### Cell culture

Canine A72 fibroblast cells (ATCC; CRL-1542) were grown in RPMI 1640 media (ATCC, Manassas, VA) supplemented with 10% inactivated Fetal Bovine Serum (Gibco, Waltham, Ma), 20mM L-glutamine (Gibco, Waltham, Ma), and 1% pen strep (Gibco, Waltham, Ma). Rat L2P41.a lung epithelial cells were grown DMEM medium (Cytiva, Marlborough, Ma.) supplemented with 10% inactivated Fetal Bovine Serum (Gibco, Waltham, Ma) and 1% pen strep (Gibco, Waltham, Ma). The L2P41.a cells were kindly provided by Dr. Tanya Miura (University of Idaho). Both A72 and L2P41.a cells were incubated in 5% CO_2_ and at 37°C. Cell media was changed every 2–3 days and cells were passaged 1:5 weekly with trypsin-EDTA 0.25% (Gibco, Waltham, Ma).

### Virus stocks

The following alphacoronaviruses were used as part of this study: Canine coronavirus (CaCoV) strain 1–71; CaCoV strain UCD-1, Feline enteric Coronavirus (FeCoV) strain WSU 79–1683, and FeCoV (FIPV) strain WSU 79–1146. CaCoV and FeCoV were chosen as they are genetically distinct (~85% sequence homology), can co-infect the same cell type (A72s), and have demonstrated evidence of recombination in nature [32]. Two betacoronaviruses were also included for comparison: murine hepatitis virus (MHV) strain JHM, and Rat coronavirus (RCoV) strain Parker. The RCoV was kindly provided by Dr. Tanya Miura (University of Idaho), while the strains of CaCoV, FeCoV, and MHV were obtained from ATCC (Manassas, VA). Strains of CaCoV and FeCoV were prepared in A72 cells; while MHV and RCoV were prepared in L2P41.a cells. All virus stocks were produced using a multiplicity of infection (MOI) of 0.01. Infections were monitored for cytopathic effects (CPE) and harvested when 100% CPE was observed (48–72 hours). Virus titers were determined using the end point dilution assay as previously described (Leibowitz et al 2011) and TCID50/ml was quantified using the TCID50 calculator tool v2.1-20-01-2017_MB (University hospital Heidelberg, Heidelberg, Germany). Virus stocks were stored at -80°C until use. Genomic sequences for each virus used in this study were deposited in GenBank (Table 1).

**Table 1:** All virus strains used in experimental co-infections and GenBank accessions of genomic sequences. Virus names and strains for all viruses used in experiments reported in this study are shown in the first two columns. Numbers in the third column denote the pairs of viruses that were co-infected together. Exact genomic sequences of virus stocks were deposited in GenBank and accessions are shown in the fourth column.

Virus	Strain	Pair	GenBank Accession
Canine coronavirus (CaCoV)	1–71	1	PP526170
Feline enteric coronavirus (FeCoV)	WSU 79–1683	1	PP526171
Canine coronavirus (CaCoV)	UCD-1	2	PP526172
Feline enteric coronavirus (FeCoV)	FIPV WSU-79-1146	2	PP526173
Mouse hepatitis virus (MHV)	JHM	3	PP526174
Rat coronavirus (RCoV)	Parker	3	PP526175

### Co-infections

A72 cells were seeded into T175 flasks (Thermo Scientific, Rochester, NY) at a concentration of 3-5x10^6^ cells/flask and left to incubate overnight. The following day, cells were co-infected at a total MOI of 1 (0.5 per virus) with CaCoV strain 1–171 and FeCoV strain WSU 79–1683. Cells and supernatant were collected into Trizol for RNA extraction at 48 hours post-infection (hpi). Samples were then sequenced using Oxford Nanopore Direct RNA Sequencing. This experiment was repeated a total of 9 times. The same experiments were then repeated using two additional strains of CaCoV and FeCoV (UCD-1 and WSU-79-1146) and also with MHV and RCoV. These additional experiments were performed in triplicate for each pair.

Using CaCoV strain 1–71 and FeCoV WSU-79-1683, additional experiments were performed under varying experimental conditions such that cells were infected at a total MOI of 0.1, 1, 2, 5, or 10 and infections were harvested either 12, 24, or 48 hpi. Chemical inactivation and Oxford Nanopore sequencing were performed as in initial experiments. Each MOI and timepoint was performed in triplicate (N = 45 experiments).

For the staggered co-infection studies, CaCoV strain 1–71 was added to A72 cells at an MOI of 2.5. Then at 2, 4, 8, and 12 hours later FeCoV was added also at an MOI of 2.5 (for a total MOI of 5). All infections were incubated for 24 hours after addition of the second virus and chemically inactivated and sequenced in the same manner as in initial experiments. Identical experiments were also performed infecting FeCoV first and CaCoV after 2, 4, 8, or 12 hours. Each experimental condition was repeated twice (N = 16 experiments).

All co-infection experiments were performed under BSL2+ conditions, meaning strict adherence to BSL2 containment but with BSL3 work practices and procedures (i.e. additional barrier gowns, N95 masks, limited access to tissue-culture room). All cultures were chemically inactivated at the conclusion of each experiment (i.e., after the designated infection time had elapsed) prior to further processing for sequencing.

### Control experiments

To validate the specificity of the algorithm on laboratory samples, A72 cells were either mock infected or singly infected with CaCoV or FeCoV at a total MOI of 2 for 48 hours. In addition, to rule out the possibility our recombinant reads were generated artificially during library preparation, we conducted a series of single CaCoV and FeCoV infections at a total MOI of 2 for 48 hours. Following infections, samples were chemically inactivated, and RNA was serially combined (to simulate a coinfection) at three points during sample preparation: (i) following the RNA extraction, (ii) following the polyA selection, and (iii) following library preparation. All samples were processed and sequenced identically as was done for co-infection experiments.

### Library preparation and sequencing

Total RNA was extracted using the guanidine thiocyanate method and polyA-tailed RNA was purified using AMPURE XP beads (Beckman Coulter). Sequencing libraries were prepared using Nanopore Direct RNA Sequencing kits and protocols (Oxford Nanopore Technologies, Oxford, UK). No polymerase chain reaction (PCR) amplification was performed at any step to eliminate the potential for PCR chimeras [80,81]. Library concentrations were evaluated using the qubit with the DNA HS kit (Invitrogen Corporation, Carlsbad, CA). Sequencing was performed on the MinION MK1C using the default settings (Oxford Nanopore Technologies, Oxford, UK). For each experiment, the sequencing was conducted for 48 hours and an entire flow cell was used per run. All sequencing runs performed in this study have been deposited in SRA (see S2 File for accession numbers).

### Bioinformatic pipeline

Basecalling and adapter removal were performed as is standard for Oxford Nanopore sequencing using Guppy (v3.1.5). Sequencing files were examined for contamination using CZ ID [44]. Reads were then subjected to BLASTn (v2.9.0) using a custom BLAST database containing only the parental virus sequences and reads with no BLAST hits were removed. Reads less than 1,000 bp were filtered out using seqkit (v0.11.0) [82]. Reads were then sorted into categories using *NanoSort*, a novel likelihood-based method developed to detect recombinant RNA, structural variant RNA, and sgRNA.

### *NanoSort* algorithm

Because CaCoV and FeCoV are ~85% identical and the error rate of Nanopore sequencing can reach 15%, simply aligning reads to parental sequences was insufficient to distinguish where a single read had both CaCoV and FeCoV origins. We therefore developed a likelihood-based algorithm called *NanoSort* that identifies recombinant reads. The code used to run *NanoSort* on viral reads is openly available at https://github.com/wellshl/NanoSort-Bonavita-Wells-Anthony-2024. *NanoSort* recombination detection is comprised of four steps: 1) split each long read into 200 bp sub-reads with a 10bp sliding window using seqkit (v0.11.0) and align each sub-read independently to the parental genomes using minimap2 (v2.23) [83]; 2) calculate the likelihood that each sub-read was sequenced from either the first or second parental virus; 3) classify the sub-read to one parent or the other based on its likelihood score (the likelihood ratio); and 4) determine if a read is recombinant or not based on the number of its sub-reads that classify to each parental virus. *NanoSort* additionally identifies structural variant RNA and sgRNA by parsing CIGAR strings to identify deletions greater than 100 bp in whole read alignments (not sliding sub-reads) with parental sequences. Reads with alignments that had junctions that corresponded to TRS locations were classified as sgRNA and those that did not were classified as structural variants.

### Estimation of Nanopore error profiles

The development of the recombination detection portion of *NanoSort* required defining the error profile of Nanopore sequencing such that we could assign probabilities to particular sequencing outcomes. These probabilities were calculated from a training dataset consisting of reads sequenced from each parental virus in unmixed samples. Reads were preprocessed and BLAST extracted as in the *Bioinformatic pipeline* section, then mapped to the respective parental genome using minimap2 (v2.23). For every position in the alignment, we recorded the target parental nucleotide as well as the nucleotides one position up- and downstream from it (*N*, a 3-mer with the target nucleotide in the center) as well as the corresponding sequenced read nucleotide, *n*. If the basecalling algorithm inserted a nucleotide, the up- and downstream parental nucleotides were recorded such that the 3-mer was designated with a gap character ‘-’ in the middle and the read nucleotide was recorded as whichever base was inserted. If a nucleotide was deleted, the parental 3-mer was unchanged and the read nucleotide was recorded as a gap. The number of occurrences for all parental 3-mers and matching sequenced nucleotides were recorded in an 80 x 5 count matrix, called *C* (S1 File). Each entry in the count matrix was then divided by the sum of its row to calculate the approximate conditional probabilities of each sequencing outcome *n* being observed given a corresponding parental nucleotide 3-mer *N* (Eq 1). For example, a T preceded by an A and followed by a G (*N* = a 3-mer of ‘ATG’) may have been sequenced and read as *n* = A, C, T, G, or a gap. The probability that the parental nucleotide T in this position was sequenced and read as a T in the read would be the number of times that this 3-mer ‘ATG’ was observed with the corresponding read nucleotide sequenced as a T divided by the total number of times the 3-mer was observed. An error profile was independently derived for each parental virus used.


$$\text{P}(n|N)=\frac{number\;of\;times\;N\;and\;n\;observed\;together}{number\;of\;times\;N\;observed}$$ (1)

### Calculation of likelihood scores

To find the likelihood that a sequence of base pairs (*n*_*1*_*…n*_*k*_) constituting an entire sub-read was generated from a given parental virus sequence (*N*_*1*_*…N*_*k*_), the probabilities of each nucleotide *n*_*i*_ having been sequenced from each corresponding 3-mer *N*_*i*_ in the parental virus sequence (P(*n*_*i*_|*N*_*i*_)) are multiplied, or alternatively for computational efficiency, the log likelihoods are summed. Log likelihoods are generated for both parental sequences using the respective parental count matrix *C*. Finally, the likelihood score *S* is calculated as the difference in log likelihoods (i.e., the likelihood ratio) for each parent (Eq 2).


$$L=\prod_{1\ldots k}\text{P}(n_{i}|N_{i})$$

$$\text{log}\left(L\right)=\sum_{1\ldots k}\;\text{log}(P(n_{i}|N_{i})$$

$$S=\text{log}{\left(L\right)}_{Parent1}-\text{log}{\left(L\right)}_{Parent2}$$ (2)

### Determination of significance of likelihood scores

Ideally, the likelihood scores for sub-reads generated from Parent 1 should all be positive and those from Parent 2 should all be negative (see Eq 2). But because the similarity of the parental sequences varies considerably across the genome, the distribution of likelihood scores also varies across the genome. Where the two parental sequences have high nucleotide identity, the likelihoods that a given read came from either parent will be similar and the likelihood score will be close to 0. In some cases, a sub-read from Parent 1 can result in a negative likelihood score, and vice versa, a sub-read from Parent 2 can result in a positive likelihood score.

In order to generate thresholds above and below which sub-reads could reliably be classified to parental viruses, we next generated distributions of likelihood scores {*S*_1_} and {*S*_2_} for each parental virus by calculating all scores for sub-reads of size 200 with a step size of 10 from a set of reads generated from an unmixed infection and mapping these scores to the genomic position of each sub-read. S5 Fig in S1 File shows this distribution of likelihood scores across the genome for CaCoV strain 1–71 and FeCoV strain WSU-79-1683 as well as their average nucleotide identity, demonstrating that likelihood scores are closer to 0 when similarity of the genomes is close to 100%. From these distributions, two classification thresholds are determined from the likelihood scores at every genomic position: 1) the threshold *t*_*1*_ above which a likelihood score *S* can be classified as Parent 1 with high confidence (positive score), and 2) the threshold *t*_*2*_ below which a likelihood score *S* can be classified as Parent 2 with high confidence (negative score). If the minimum value of the set {*S*_*1*_} was above 0 and the maximum value of the set {*S*_*2*_} was below 0, both *t*_*1*_ and *t*_*2*_ were set to 0. If the minimum value of the set {*S*_*1*_} was less than 0, *t*_*1*_ was set as the maximum value of {*S*_*2*_} or 0, whichever was greater. If the maximum value of the set {*S*_*2*_} was greater than 0, *t*_*2*_ was set as the lesser of either the minimum value of {*S*_*1*_} or 0. In cases where *t*_*1*_ > *t*_*2*_, any scores found to fall between *t*_*1*_ and *t*_*2*_ were left unclassified (S6 Fig in S1 File). In order for a read to be called recombinant, at least 31 of its sub-reads (totaling 500 bp) must be classified to each parental virus using these thresholds (the choice of a 500bp threshold is discussed in the following section).

### Validation of recombination detection

Validation of the ability of *NanoSort* to detect recombinant reads was performed by simulating recombinant CaCoV strain 1–71 / FeCoV strain WSU-79-1683 reads *in silico*. Two recombinant reads were simulated with a breakpoint at every genomic position: one starting with FeCoV and one starting with CaCoV. The first 700bp were derived from the first parent and the subsequent 700bp were derived from the second, resulting in a single recombinant read of size 1,400bp with a breakpoint exactly in the center. A size of 700bp was chosen because 500bp from each parent was the minimum required for a read to be called recombinant. Finally, each parental portion of the simulated recombinant read was mutated according to probabilities derived from the trained Nanopore error profiles. Simulated recombinant reads were then analyzed with *NanoSort*. Sensitivity across the genome was calculated using a sliding window of size 100 and a step size of 10. Within each window, sensitivity was calculated by dividing the number of reads with breakpoints within the window that were successfully classified as recombinant divided by the total number of simulated reads with breakpoints within the window. Overall sensitivity was 98.6% and generally exceeded 95% across the entire genome (S2 Fig in S1 File). Sensitivity drops off significantly at the 3’ end of the genome due to overlapping distributions of {*S*_*1*_} and {*S*_*2*_} in this region (see S5 Fig in S1 File and *Determination of significance of likelihood scores*).

We also validated the potential for *NanoSort* to identify false positive recombinants by running the algorithm on negative controls, where viruses were grown independently and combined immediately before sequencing (see *Control Experiments* above). Where the distributions of *{S1}* and *{S2}* become close to 0, in most cases the algorithm will assign a sub-read as “unclassified”; however, in some cases the algorithm can incorrectly classify a sub-read as the opposite parent. To overcome the potential for false positive recombination events to be called after misclassification of sub-reads, we implemented a 500bp minimum (totaling 31 sliding sub-reads) to be classified to each parent before a read could be called recombinant. This threshold was chosen to balance the minimization of false positives in negative controls and maximize dectection of true positives in simulated positive controls and experimental replicates.

### SIR model for predicting rates of co-infection

To generate predictions about rates of recombination across different MOIs and at varying time points, we modeled the *in vitro* dynamics of infection using a discrete-time Susceptible-Infectious-Removed (SIR) model. A Jupyter notebook containing the code used to produce this model is openly available at doi.org/10.5281/zenodo.10854671. We assumed that both viruses grow at identical rates, that infection events are independent, and that a cell ‘bursts’ (even though coronaviruses are non-lytic) and is removed from the system after one replication cycle of being infected. We parameterized a small ‘birth’ rate for susceptible cells at a rate of 0.5 births per cell per cycle and began each time series with 10^6^ susceptible cells (*S*(0)). We estimated the fraction of cells infected at onset (*I*(0)) for a given initial *MOI*(0) as equal to the probability of a single cell being infected with at least two virions. Using a Poisson distribution parameterized with *λ* = *MOI*(0), the probability a single cell is infected with at least two virions is equal to 1 minus the probability that a given cell is infected with one or fewer virions (1 − *P*(*x* ≤ 1)). We also estimate the fraction of co-infected cells as the probability of a single cell being independently infected with at least two virions of each virus, (1 − *P*(*x* ≤ 1))^2^, with the Poisson distribution parameterized with *λ* = *MOI*(0)/2. Note that the SIR model does not include a co-infected category; these cells are accounted for within the “Infected” category and are simply recorded as a separate calculation at each step. Each discrete time step represented approximately one viral replication cycle (~12 hours). MOI was measured as a function of TCID_50_ for our experimental studies, so this value was converted to approximate plaque forming units by multiplying by 0.69.

At each time *t*, the number of cells infected in the previous step *I*(*t-1*) release a set number of infectious virions (the ‘burst size’) before being removed from the system. The burst size was parameterized at 1000 virions [84]. The number of previously infected cells multiplied by the burst size and divided by the number of remaining susceptible cells is used to calculate MOI at time *t*, from which a new fraction of susceptible cells is estimated to be infected at the new time step according to a Poisson distribution with *λ* = *MOI*(*t*) The fraction of co-infected cells is also estimated with a Poisson distribution and *λ* = *MOI*(*t*)/2 The model is iterated across 6 replication cycles or ~72 hours. The number of susceptible *S*(*t*), infected *I*(*t*), co-infected *co* − *I*(*t*), and removed *R*(*t*) cells at each iteration was calculated with the following equations with P_1_~Poisson(*λ* = *MOI*(*t*)) and P_2_~Poisson(*λ* = *MOI*(*t*)/2):

$$MOI\left(t\right)=\frac{I\left(t-1\right)*burst\;size}{S\left(t-1\right)}$$

$$S\left(t\right)=S\left(t-1\right)-\left(1-P\left(x\leq 1\right)\right)*S\left(t-1\right)+birth*S\left(t-1\right)$$

$$I\left(t\right)=\left(1-P_{1}\left(x\leq 1\right)\right)*S\left(t-1\right)$$

$$co-I\left(t\right)={\left(1-P_{2}\left(x\leq 1\right)\right)}^{2}*S\left(t-1\right)$$

$$R\left(t\right)=I\left(t-1\right)$$

## Supporting information

S1 File Contains all supplementary figures and tables. (DOCX)

S2 File Contains all read count data and rates for sgRNA, dRNA, and recombinant RNA formation for all experimental co-infections reported here. (XLSX)
