# Supplementary material for: Cellular dynamics shape recombination frequency in coronaviruses
Source: PLoS Pathog. 2024 Sep 27;20(9):e1012596. doi: 10.1371/journal.ppat.1012596 (PMC11463787; doi:10.1371/journal.ppat.1012596)
Supplement: S1 File — (DOCX) [file ppat.1012596.s001.docx]

**Supplemental Figures and Tables**

**
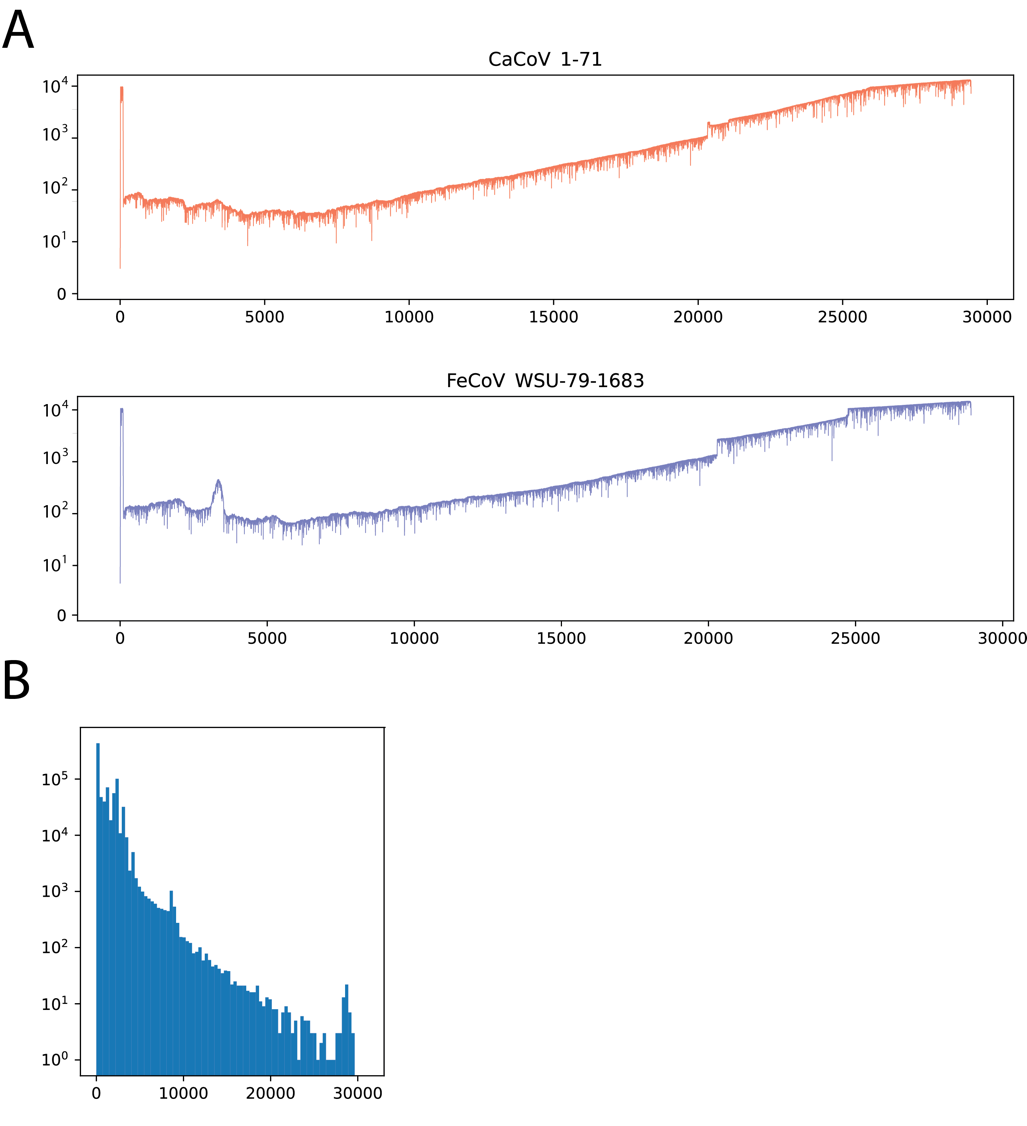
**

*Figure S1. Observed coverage of viral genomes and histogram of viral read lengths for a single coinfection experiment.* (A) Read coverage of CaCoV 1-71 and FeCoV WSU-79-1683 observed at 48 hpi in a single co-infection experiment (Replicate 7). Y-axis denotes number of reads (log scale), and x-axis denotes genomic position. Average read depth for this sequencing run was 1,911X for CaCoV and 2,007X for FeCoV. (B) Histogram of viral read lengths for both viruses for the same experiment. The averge read length was 1,954bp. Y-axis denotes number of reads (log scale), and x-axis denotes read length. All 9 replicates of this initial experiment yielded similar results.

**
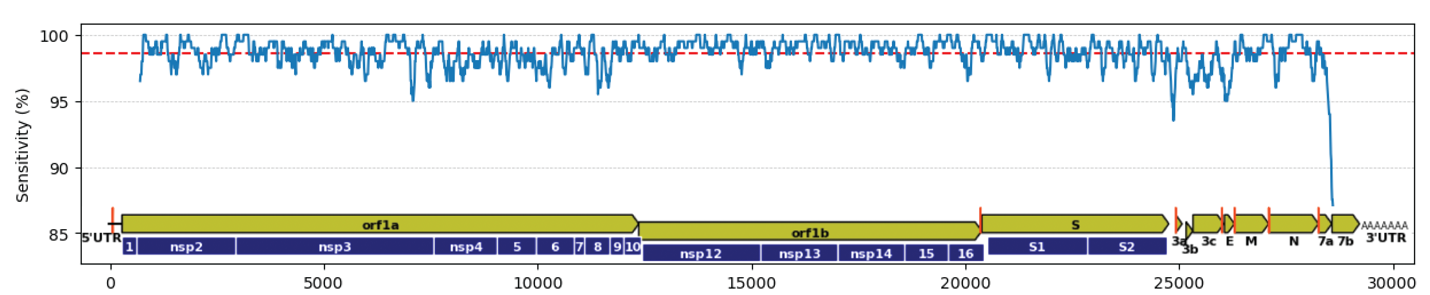
**

*Figure S2*. *Sensitivity of recombination detection using simulated recombinant reads*. The sensitivity of the recombination detection algorithm *NanoSort* calculated using a sliding window with size 100 and step size 10. The dashed red line indicates the average sensitivity of 98.6%. Yellow polygons at the bottom indicate gene boundaries and blue polygons indicate the boundaries of each non-structural protein (nsp) within orf1ab as well as the S1 and S2 subunits of the spike gene.


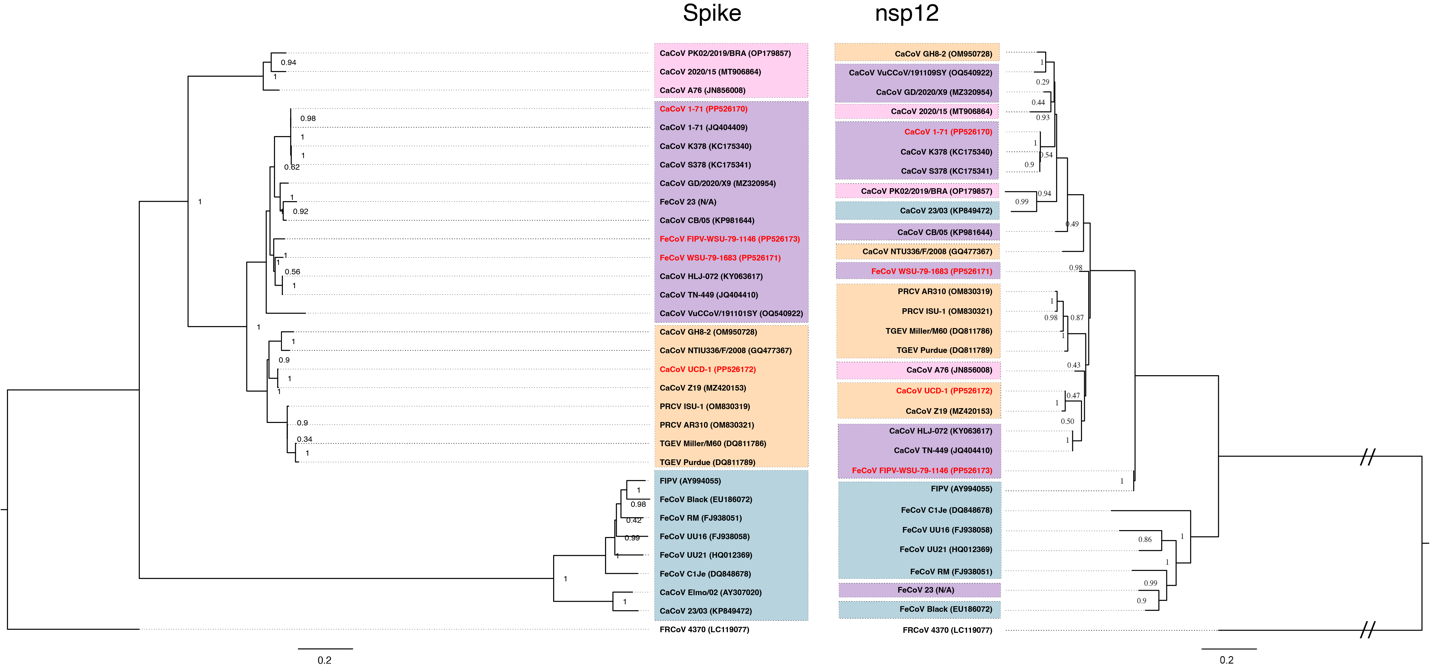


*Figure S3. Phylogenetic trees for nsp12 and the spike gene of selected Canine coronavirus and Feline coronavirus strains.* Maximum likelihood phylogenetic trees for nsp12 and the spike gene were generated using MEGA X v10.2.6 using a GTR substitution model with gamma-distributed rates across sites and 500 bootstraps. Tips are highlighted with colored boxes designating the different serotypes: blue: serotype 1; purple: serotype 2a, orange: serotype 2b, pink: serotype 2c,. Differences in topology between nsp12 and the spike gene are evidence of historical recombination events.

*
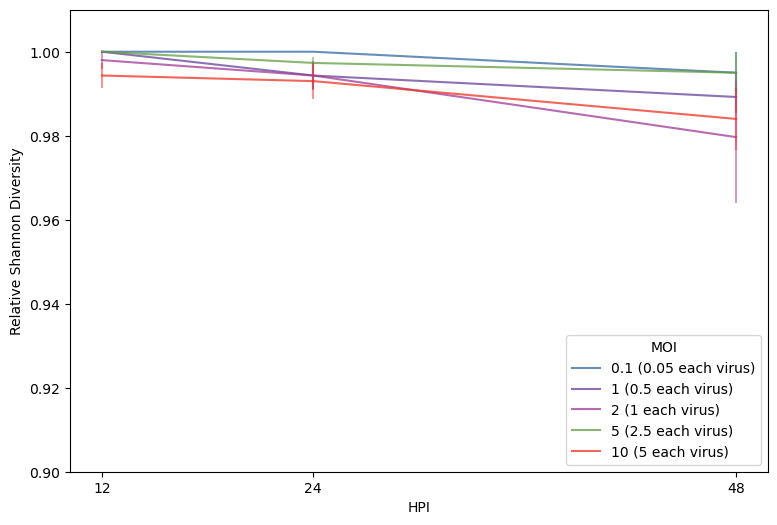
*

*Figure S4. Change in relative Shannon diversity score across experiment times and starting MOIs.* Relative Shannon diversity score was calculated as the observed Shannon diversity score divided by the maximum possible Shannon diversity score (obtained when the proportions of all observed recombinants in an experiment were equal). Experimental co-replication times are shown on the *x*-axis and relative diversity scores on the *y*-axis. Starting MOI is indicated by different colored lines. Vertical bars show the standard error for the triplicate experiments at each point.

**
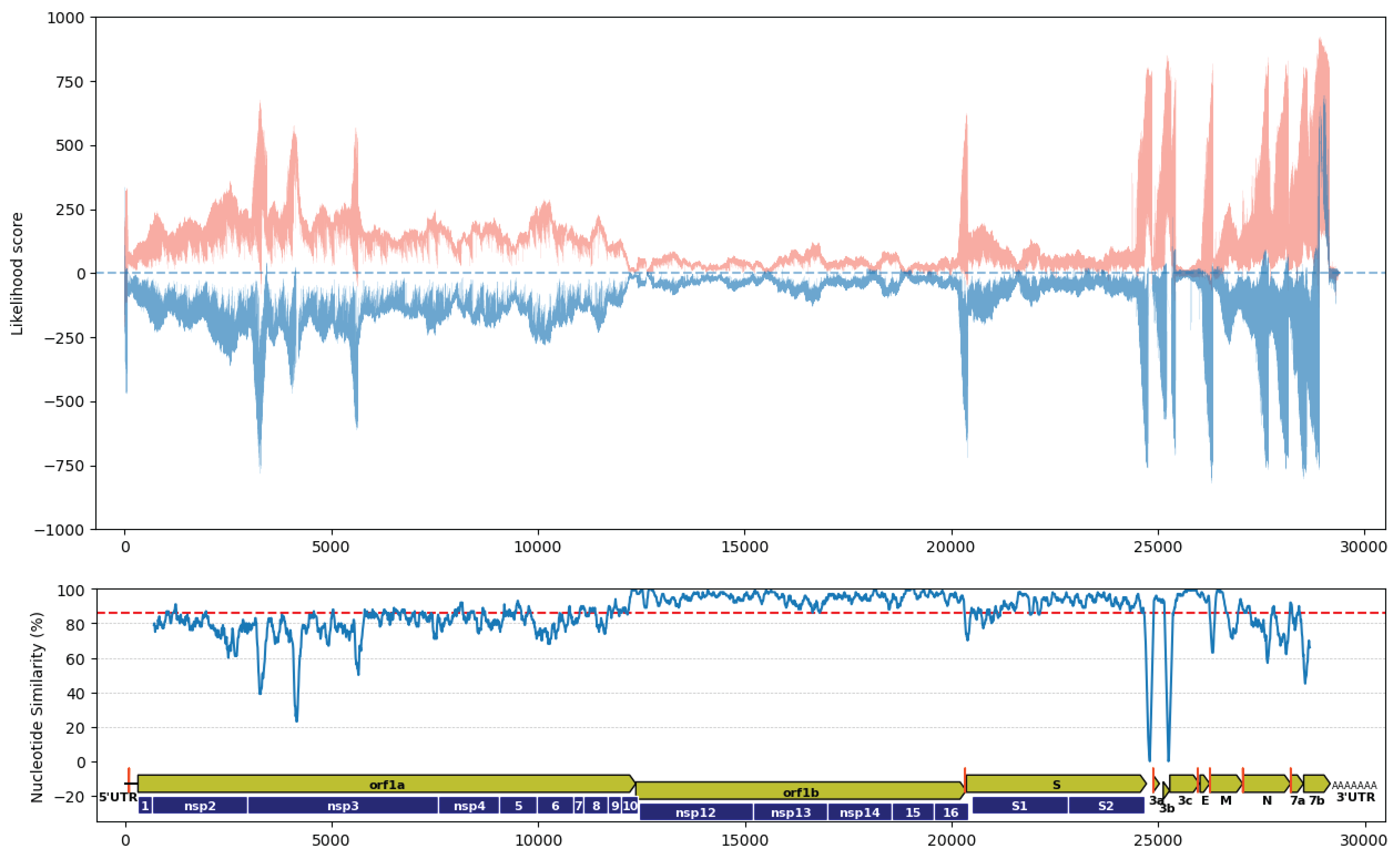
**

*Figure S5*. *Distribution of likelihood scores for each parental virus and average nucleotide identity*. The top panel shows computed likelihood scores for sub-reads generated from CaCoV strain 1-71 (red) and FeCoV strain WSU-79-1683 (blue). The bottom panel shows the nucleotide identity between CaCoV and FeCoV on a sliding window scale of size 100 and step size 10. The dashed red line represents the average overall nucleotide, which is ~85%. Yellow polygons at the bottom indicate gene boundaries and navy blue polygons indicate the boundaries of each non-structural protein (nsp) within orf1ab as well as the S1 and S2 subunits of the spike gene.


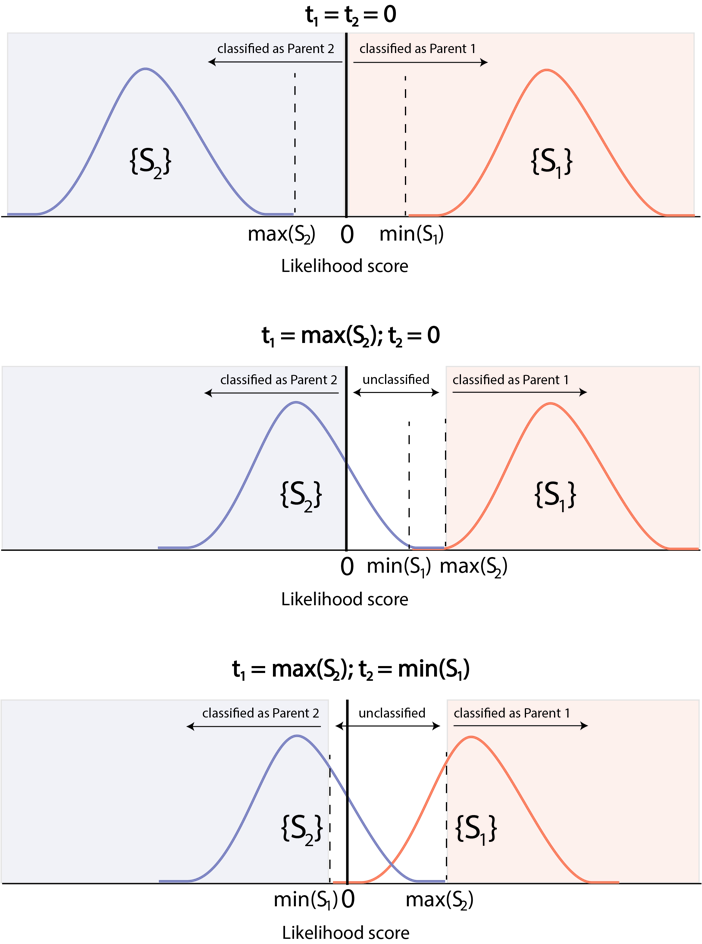


*Figure S6. Determination of cutoff thresholds for classification of sub-reads based on likelihood scores.* In order to be classified as Parent 1, a sub-read must have a likelihood score greater than *t_1_*. To be classified as Parent 2, it must have a score less than *t_2_*. The thresholds *t_1_* and *t_2_* are obtained from the minimum and maximum of the distributions of scores calculated for sub-reads from each parent at each genomic position, {*S_1_*} and {*S_2_*}, respectively. Where *t_1_* > *t_2_*, reads falling in between thresholds remain unclassified.

*Table S1*. *Example of a count matrix C*. This table shows a truncated count matrix for all parental nucleotide 3-mers beginning with the nucleotide ‘A’ (20 of 80 total rows). The number of events counted for each parental 3-mer (rows) and sequenced nucleotide (columns) pair is shown in the count column, and the calculated conditional probability of observing the sequenced nucleotide *n* given the parental 3-mer *N*, $P(n|N)$, is also shown.

|  | **A** | | **T** | | **C** | | **G** | | **-** | |
| --- | --- | --- | --- | --- | --- | --- | --- | --- | --- | --- |
|  | Count | P(n\|N) | Count | P(n\|N) | Count | P(n\|N) | Count | P(n\|N) | Count | P(n\|N) |
| **AAA** | 34495096 | 96.66% | 209811 | 0.59% | 55925 | 0.16% | 161833 | 0.45% | 765393 | 2.14% |
| **AAT** | 24292818 | 96.14% | 157709 | 0.62% | 100192 | 0.40% | 169044 | 0.67% | 547465 | 2.17% |
| **AAC** | 22197447 | 94.75% | 437761 | 1.87% | 40874 | 0.17% | 35304 | 0.15% | 715388 | 3.05% |
| **AAG** | 27012412 | 97.44% | 82546 | 0.30% | 11216 | 0.04% | 237198 | 0.86% | 379608 | 1.37% |
| **ATA** | 41024 | 0.31% | 12364454 | 92.11% | 135213 | 1.01% | 19195 | 0.14% | 863814 | 6.43% |
| **ATT** | 257136 | 0.83% | 27256427 | 88.48% | 538234 | 1.75% | 33312 | 0.11% | 2721531 | 8.83% |
| **ATC** | 231569 | 1.43% | 14929057 | 92.12% | 92591 | 0.57% | 53136 | 0.33% | 900620 | 5.56% |
| **ATG** | 29917 | 0.15% | 18542637 | 95.47% | 313032 | 1.61% | 25486 | 0.13% | 511777 | 2.63% |
| **ACA** | 36980 | 0.14% | 470875 | 1.78% | 24794611 | 93.83% | 32999 | 0.12% | 1089878 | 4.12% |
| **ACT** | 63947 | 0.38% | 616912 | 3.66% | 15228742 | 90.45% | 24001 | 0.14% | 902853 | 5.36% |
| **ACC** | 19687 | 0.18% | 285679 | 2.59% | 9987489 | 90.41% | 7339 | 0.07% | 746679 | 6.76% |
| **ACG** | 2875 | 0.05% | 136111 | 2.21% | 5910554 | 95.94% | 5041 | 0.08% | 106360 | 1.73% |
| **AGA** | 29157 | 0.10% | 28699 | 0.10% | 17561 | 0.06% | 28718753 | 98.36% | 402973 | 1.38% |
| **AGT** | 31313 | 0.20% | 32352 | 0.21% | 11009 | 0.07% | 15455029 | 98.15% | 216348 | 1.37% |
| **AGC** | 77086 | 0.67% | 24669 | 0.22% | 7688 | 0.07% | 11086034 | 96.74% | 263712 | 2.30% |
| **AGG** | 165775 | 1.15% | 33762 | 0.23% | 12925 | 0.09% | 13291775 | 91.81% | 972845 | 6.72% |
| **A-A** | 118090 | 13.74% | 358198 | 41.66% | 244382 | 28.42% | 139082 | 16.18% | n/a | n/a |
| **A-T** | 262808 | 12.55% | 1364323 | 65.16% | 297191 | 14.19% | 169367 | 8.09% | n/a | n/a |
| **A-C** | 127427 | 8.16% | 949423 | 60.83% | 429389 | 27.51% | 54460 | 3.49% | n/a | n/a |
| **A-G** | 137085 | 18.22% | 145480 | 19.33% | 103300 | 13.73% | 366614 | 48.72% | n/a | n/a |
